# Flexible Nasendoscopy in Stevens–Johnson Syndrome/Toxic Epidermal Necrolysis: A Ten-Year Otolaryngology Experience

**DOI:** 10.3390/medicina61030513

**Published:** 2025-03-16

**Authors:** Matthew Min Xian Yii, Antonia Rowson, Milan van Ammers, Jessica Prasad

**Affiliations:** 1 Ear, Nose and Throat Department, Alfred Health, Melbourne, VIC 3004, Australia; 2 Eastern Health, Box Hill, VIC 3128, Australia

**Keywords:** Stevens–Johnson syndrome, toxic epidermal necrolysis, flexible nasendoscopy, airway

## Abstract

*Background and Objectives*: The primary objective of this study was to identify factors predictive of laryngeal involvement in patients with Stevens–Johnson syndrome/toxic epidermal necrolysis (SJS/TEN). The secondary objective was to observe the effect of laryngeal involvement upon short-term patient prognosis, including intensive care unit (ICU) stay and intubation rates. We present the largest cohort of patients examined for upper aerodigestive manifestations of SJS/TEN. *Materials and Methods*: We performed a retrospective observational analytic study of patients at a state-wide Australian Burns referral center between January 2013 to December 2022. Inclusion criteria were adult patients who underwent flexible nasendoscopy (FNE) with biopsy-proven SJS/TEN. Data collected from medical records included patient factors, aerodigestive symptoms, bedside examination, FNE findings, TEN-specific severity-of-illness score (SCORTEN) on admission, and patient outcomes such as intubation and ICU admission. *Results*: Fifty-four patients with biopsy-proven SJS/TEN underwent FNE, with 17 (31.5%) identified to have laryngeal involvement. Laryngeal involvement was not significantly associated with intubation, ICU stay, or mortality (*p* > 0.05). The presence of either aerodigestive symptoms or oral cavity involvement was highly sensitive (94.1%, 95% CI 73.0–99.7%) for laryngeal involvement. *Conclusions*: We did not find laryngeal involvement in SJS/TEN to significantly impact short-term outcomes, including intubation or mortality. FNE is the gold standard of upper aerodigestive assessment. Simple clinical evaluation of the oral cavity and a history of aerodigestive symptoms also provided a sensitive predictor of the laryngeal complications of SJS/TEN.

## 1. Introduction

Stevens–Johnson syndrome (SJS) and toxic epidermal necrolysis (TEN) exist on a spectrum of rare severe cutaneous immune-mediated adverse reactions in which extensive epidermal necrosis and mucositis occur [1]. SJS and TEN represent the same disease process but are differentiated by the extent of body surface area (BSA) involvement; SJS involves less than 10% BSA, SJS/TEN overlap 10–30% BSA, and TEN involves more than 30% BSA. The terms “SJS/TEN” and “epidermal necrolysis” encompass the spectrum of disease. This severe adverse reaction is usually triggered by medication [2], with release of cytotoxic proteins by drug-specific CD8+ T cells causing epidermal necrolysis [1].

An estimated incidence of five to six cases per million per year suffer SJS/TEN [3]. Female gender, increasing age, human immunodeficiency virus infection, connective tissue disease, malignancy, and certain human leukocyte antigen phenotype frequencies are risk factors for this disease [3,4]. Where medications are not causative, other precipitants include mycoplasma pneumonia and vaccination. A further 15–20% of cases remain idiopathic.

Widespread epidermal and mucosal involvement merits a routine multidisciplinary approach. Mucosal surfaces of the oral cavity (90%), nasal cavity (50%), ear (50%), and larynx (30%) are commonly affected [5]. Aerodigestive mucosal involvement may compromise the airway via oedema, hypersecretion, and inflammation, necessitating definitive airway management [6]. Otolaryngology input may be sought to assess upper aerodigestive involvement not assessable simply by anterior rhinoscopy or oral cavity examination. Flexible nasendoscopy (FNE) can be performed at the bedside with topical anesthetic to assess the larynx and hypopharynx [7]. FNE is the gold standard for diagnosis of laryngeal involvement of SJS/TEN; however, predictive factors for laryngeal involvement and the utility of assessment in predicting intubation are unknown [6]. Limited primary literature exists regarding the acute management of aerodigestive SJS/TEN and its impact upon a patient’s admission.

The primary objective of this study is to determine factors predictive of laryngeal involvement. The secondary objective is to determine the influence of laryngeal involvement on patient prognosis, intensive care unit (ICU) stay, and rates of intubation.

## 2. Materials and Methods

We performed a retrospective observational analytic study of patients at a state-wide Australian burns referral center, drawing patients from a ten-year period between January 2013 and December 2022. Inclusion criteria were for patients at least 18 years of age at time of admission, who had a confirmed histologic diagnosis of SJS/TEN, and had undergone a FNE examination during their admission. Ethical approval was granted by the Alfred Health Research and Ethics Committee (approval number: 674/22).

Patients were identified via ear, nose, and throat (ENT) and burns unit audit data. Data were collated into a de-identified spreadsheet accessible only by researchers approved by the Alfred Health Research and Ethics Committee. Information gathered from medical records included age at time of admission, time from presentation to FNE, causative agent of SJS/TEN, total body surface area involved, area of desquamation, and TEN-specific severity-of-illness score (SCORTEN) on admission. Data points of aerodigestive assessment findings collected were dysphonia, stridor, dysphagia/odynophagia, and the presence of lip, oral cavity, or nasal cavity involvement. FNE findings collected were involvement of the nasopharynx, oropharynx, and/or larynx, and descriptors of subsites of these regions if involved. In patients who underwent repeat FNE, we documented whether findings worsened compared to initial assessment. Outcome data points collected were whether patients required intubation, cause for intubation, whether patients were admitted to ICU, length of ICU stay, length of hospital stay, and mortality.

SCORTEN is a validated severity-of-illness score applicable to patients with SJS/TEN [8]. It is calculated using seven prognostic factors, each scoring one point:

1. Age ≥ 40 years;
2. Heart rate ≥ 120 beats per minute;
3. Associated malignancy;
4. Detached body surface area ≥ 10%;
5. Serum urea > 10 mmol/L;
6. Serum bicarbonate < 20 mmol/L;
7. Serum glucose > 14 mmol/L.

Mortality rates increase with SCORTEN; a score ≥ 5 indicates a mortality rate of >90%. The majority of cases had SCORTEN documented on admission. For scores not explicitly documented, this was calculated at time of data collection from admission electronic medical record data and historical pathology results.

Statistical analysis was performed using GraphPad Prism 9. All continuous variables were found to be non-normally distributed on D’Agostino–Pearson testing. When comparing categorical variables with categorical outcomes (laryngeal involvement vs. intubation/ICU admission/mortality), Fisher’s exact test was used due to the small sample size. The non-parametric Mann–Whitney U testing was used to determine categorical variables with continuous outcomes (laryngeal involvement vs. ICU/hospital length of stay). Sensitivity and specificity values with a 95% confidence interval (CI) were calculated using the Wilson/Brown method. Simple logistic regression was used to compare SCORTEN with the presence of laryngeal involvement. Statistical significance was set at *p* < 0.05.

## 3. Results

Sixty-seven adult patients were identified with biopsy-proven SJS/TEN over the relevant ten-year period. Fifty-four adult patients underwent FNE. Of the thirteen excluded patients, six were not referred to ENT, five were not scoped as they were intubated prior to review, one patient died prior to review, and one patient was assessed by ENT, but no FNE was performed.

The median age of patients meeting inclusion criteria was 57.5 (IQR 33.5–68.3) years (Table 1). Twenty-five patients (46.3%) were male, and 29 (53.7%) were female. The most common precipitants were antibiotics in twenty cases (37.0%), unknown cause in eleven (20.4%), and antiepileptics in seven (13.0%). The median time from either emergency department presentation or initial documented suspicion of SJS/TEN (if admitted prior to developing SJS/TEN) to FNE was 10.5 (IQR 2.8–24.0) hours. On admission/initial review for SJS/TEN, the median total body surface area of SJS/TEN was 40.0% (IQR 30.0–70.0%) and median body surface area of detachment was 11.0% (IQR 5.0–25.8%). SCORTEN 2 was the most common, accounting for 17 patients (31.5%) at initial review. At time of initial ENT review, upper aerodigestive signs/symptoms were described in 29 (53.7%) cases. Dysphagia/odynophagia was present in twenty-four (44.4%) patients, and hoarseness of voice was documented in nine (16.7%) patients. No patients were documented to have stridor on review.

Twenty-one (38.9%) patients required intubation during their admission (not including patients who were intubated, then immediately extubated for operative care). The indications for intubation were to facilitate operative intervention in nine patients, for upper airway compromise in five patients, impaired consciousness in three patients, respiratory distress in two patients, and agitation in two patients. Thirty-six (66.7%) patients required ICU admission. For patients admitted to ICU, the median length of stay was 6.5 days (IQR 3.0–14.8). The median length of hospital stay following presentation/initial suspicion of SJS/TEN was 13 (IQR 8.8–19.3) days. Six (11.1%) patients died during their admission.

Forty-one (75.9%) patients suffered oral cavity involvement, with lips being the most commonly involved subsite in thirty-three (61.1%) cases. Sixteen (29.6%) were found to have nasal cavity involvement. FNE examination found three patients (5.6%) to have nasopharyngeal mucosal involvement. Sixteen (29.6%) were documented to have oropharyngeal involvement, most commonly in the posterior pharyngeal wall (fourteen cases, 25.9%). Seventeen patients (31.5%) were identified to have laryngeal involvement, with the supraglottis most commonly implicated (sixteen cases, 29.6%). Only two cases (3.7%) had documented vocal cord involvement.

No significant correlation was found between laryngeal involvement and ICU admission, intubation, or mortality on Fisher’s exact test statistical analysis (Table 2). Mann–Whitney U testing demonstrated no significant difference in ICU length of stay between patients with (median 3.0 days) and without (median 3.0 days) laryngeal involvement (U = 296, *p* = 0.7309). Similarly, there was no significant difference in the hospital length of stay between patients with (median 12.0 days) and without (median 13.0 days) laryngeal involvement (U = 294, *p* = 0.7084). No significant association was established between SCORTEN and laryngeal involvement. On simple logistic regression modelling, for each increase in SCORTEN grade, the odds of laryngeal involvement were multiplied by 0.9 (95% CI 0.6–1.4).

Simple oral cavity examination appeared to be an effective screening tool for laryngeal involvement, with oral cavity involvement of any subsite having a sensitivity of 88.2% (95% CI 65.7–97.9%) and specificity of 29.7% (95% CI 17.5–45.8%); aerodigestive signs/symptoms (odynphagia/dysphagia, hoarse voice, or stridor) had a sensitivity of 76.5% (95% CI 52.7–90.4%) and specificity of 29.7% (95% CI 17.5–45.8%) for laryngeal involvement; a combination of either oral cavity involvement or aerodigestive symptoms had a sensitivity of 94.1% (95% CI 73.0–99.7%) and specificity of 29.7% (95% CI 17.5–45.8%) for laryngeal involvement.

## 4. Discussion

This study represents the largest cohort of cases examining ENT manifestations of SJS/TEN in the literature [5,6,9,10]. The median age (57.5), gender (53.7% female, 46.3% male), BSA of detachment (11.0%), and total BSA involved (40.0%) were comparable to other studies [5,6,9,10]. Median SCORTEN 2 on admission and the rate of intubation (37.0%) were also similar to previously examined patient cohorts. Five patients were excluded from this review due to intubation precluding them from FNE examination. Resultantly, our study population may have lower body surface area involvement and SCORTEN by not including patients who likely had more severe disease.

Aerodigestive symptoms and signs should prompt urgent FNE to evaluate the upper airway. Despite this, we did not find any significant association between laryngeal involvement and intubation, ICU admission, or mortality. This reflects findings from similar cohorts where FNE findings were not associated with intubation [6]. Whilst this finding does not confer the risk of upper airway compromise from laryngeal involvement, it reflects that a multi-system disease such as SJS/TEN may necessitate intubation for a variety of indications. The most common reason for intubation in our cohort was to facilitate operative intervention, as patients were often subject to multiple procedures. Similarly, we did not identify statistically significant differences in length of stay in ICU or hospital for patients with laryngeal involvement. Again, considering the multi-system nature of SJS/TEN, upper aerodigestive manifestations may only represent a small part of the morbidity of this disease process. Despite no significant correlations between laryngeal involvement and ICU admission, length of stay, or intubation, the upper airway status should be considered in multidisciplinary discussions on a case-by-case basis when making decisions regarding intubation and ward or ICU disposition.

Identifying laryngeal involvement in SJS/TEN patients may confer other benefits; involvement may predict rates of pulmonary complications (including oedema, atelectasis, pneumonia), and the potential for long-term voice and swallowing impairment may be recognized [5,9]. For instance, Glasson et al. described four patients who required endoscopic surgical management of hypopharyngeal synechiae in the months following initial management of TEN [9].

FNE allows for definitive diagnosis of laryngeal involvement. Our results suggest that history and simple oral cavity examination are highly sensitive for laryngeal involvement. In our study, there was only a single case with laryngeal involvement on FNE without aerodigestive symptoms or oral cavity ulceration. The patient did not require intubation, and there was no progression of disease on serial examination. The absence of oral cavity involvement and aerodigestive signs and symptoms may reassure clinicians that laryngeal involvement is unlikely.

This study is limited by its retrospective nature. The rarity of SJS/TEN makes prospective investigation difficult. Further, a lack of standardized protocol resulted in variable time from presentation to FNE and inconsistency with repetition of FNE examination. The five patients that did not receive FNE, as they were intubated prior to ENT review, would have had a high probability of having laryngeal involvement, creating selection bias for our cohort meeting inclusion criteria. In addition, the study only observed short-term sequelae of aerodigestive SJS/TEN. Aerodigestive mucosal involvement may impact long-term voice and swallowing outcomes; this was not assessed in our study and should be addressed further in future research endeavors. Further prospective study with standardized routine nasendoscopy may provide further information of clinical progression or treatment response of aerodigestive SJS/TEN following initial diagnosis.

## 5. Conclusions

We did not establish a direct correlation between laryngeal involvement and clinical outcomes including intubation, ICU admission, or mortality in SJS/TEN patients. Whilst FNE remains the gold standard for diagnosing upper aerodigestive involvement, our findings suggest that history-taking and thorough oral cavity examinations are highly sensitive for detecting laryngeal involvement in patients that are not already intubated. Further research into the long-term outcomes of upper aerodigestive SJS/TEN may also help identify associated risks of chronic voice or swallow impairment.

## Figures and Tables

**Table 1:** Descriptive statistics.

Patient Demographics	Age (years)	median (IQR)	57.5 (33.5–68.3)
	Sex (male)	*n (*%)	25 (46.3%)
	Sex (female)		29 (53.7%)
SJS/TEN Factors	Total body surface area involved	median (IQR)	40.0% (30.0–70.0)
	Body surface area of desquamation		11.0% (5.0–25.8)
	SCORTEN		
	0	*n* (%)	6 (11.1%)
	1		15 (27.8%)
	2		17 (31.5%)
	3		9 (16.7%)
	4		4 (7.4%)
	≥5		3 (5.6%)
Aerodigestive Assessment Findings	Aerodigestive signs/symptoms	*n* (%)	29 (53.7%)
	Odynophagia/dysphagia		24 (44.4%)
	Hoarse voice/dysphonia		9 (16.7%)
	Stridor/shortness of breath		0 (0.0%)
	Oral cavity involvement		41 (75.9%)
	Nasal cavity involvement		16 (29.6%)
	Nasopharynx involvement		3 (5.6%)
	Oropharyngeal involvement		16 (29.6%)
	Posterior pharyngeal wall		14 (25.9%)
	Base of tongue		7 (13.0%)
	Laryngeal involvement		17 (31.5%)
	Supraglottis		16 (29.6%)
	Glottis		2 (3.7%)

**Table 2:** Subgroup analyses of laryngeal involvement vs. outcomes.

	Laryngeal Involvement *n* (%)	No Laryngeal Involvement *n* (%)	Odds Ratio (95% CI)	*p*-Value
ICU admission No ICU admission	11 (64.7%)	25 (67.6%)	0.9 (0.3–3.0)	>0.9999
	6 (35.3%)	12 (32.4%)		
Intubation No intubation	8 (47.1%)	13 (35.1%)	1.6 (0.6–4.8)	0.5490
	9 (52.9%)	24 (64.9%)		
Mortality No mortality	2 (11.8%)	4 (10.8%)	1.1 (0.2–5.0)	>0.9999
	15 (88.2%)	33 (89.2%)		

Odds ratios were calculated via Fisher’s exact test.

## Data Availability

The data presented in this study are available on request from the corresponding author due to the risk of patient identification in a cohort suffering a rare disease.

## Abbreviations

The following abbreviations are used in this manuscript:

SJS	Stevens–Johnson syndrome
TEN	toxic epidermal necrolysis
SJS/TEN	Stevens–Johnson syndrome/toxic epidermal necrolysis
BSA	body surface area
FNE	flexible nasendoscopy
ICU	intensive care unit
ENT	ear, nose, and throat
SCORTEN	TEN-specific severity-of-illness score
mmol/L	millimoles per liter
CI	confidence interval
